# Being slightly overweight is associated with a better quality of life in breast cancer survivors

**DOI:** 10.1038/s41598-018-20392-3

**Published:** 2018-02-14

**Authors:** Juan Xia, Zheng Tang, Qinglong Deng, Jiwei Wang, Jinming Yu

**Affiliations:** 1 Institute of Clinical Epidemiology, Key Laboratory of Public Health Safety, Ministry of Education, Key Lab of Health Technology Assessment of Ministry of Health, , School of Public Health, Fudan University, 130 Dong-An Road, Shanghai, 200032 China; 2 Department of Liver Surgery, Liver Cancer Institute, Zhongshan Hospital, Fudan University; Key Laboratory of Carcinogenesis and Cancer Invasion of Ministry of Education, Shanghai, China

## Abstract

To examine the association between BMI and QOL in breast cancer survivors in China, we conducted a cross-sectional survey and recruited 10708 breast cancer survivors. Survivors self-reported QOL was measured using the EORTC QLQ-C30 and the QLQ-BR23. The impact of BMI on QOL was examined through standard least squares regression. Normal weight and overweight survivors were more likely to have a better QOL than underweight and obese survivors and the results were similar to survivors diagnosed as having chronic diseases. After adjustment for clinical and sociodemographic factors, the QOL increased with increasing BMI in breast cancer survivors ranged from underweight to overweight with no chronic diseases, especially in the scales of emotional function and fatigue. Obese breast cancer survivors reported a significantly worse QOL compared to normal weight and overweight breast cancer survivors. Within breast cancer survivors with one or more chronic diseases, it was more obvious that overweight ones had a significantly better QOL with clear evidence of a dose relationship across underweight to overweight in almost all scales. Unlike obese breast cancer survivors without chronic diseases, the ones with chronic disease(s) had a similar QOL compared to normal weight breast cancer survivors in all scales except in the domain of fatigue.

## Introduction

The increasing prevalence of overweight and obesity has become a major threat to public health^1^ and the World Health Organization (WHO) listed obesity as one of today’s most blatantly visible yet most neglected public health problems and dubbed the global epidemic “globesity”^2^. Indeed, overweight and obesity are well-known risk factors that can cause and aggravate a number of chronic diseases^3,4^ including cardiovascular diseases^5^, certain forms of cancer^6,7^, and metabolic diseases^8^. Nevertheless, substantial data have shown a survival benefit of overweight and moderate obesity in patients with chronic diseases^9–11^, a better outcome was seen in the overweight and moderately obese patients compared with the normal weight patients^12–15^.

Breast cancer is the most prevalent malignant cancer in China, accounting for 15% of all female primary cancers^16^, and has a significant upward trend in the incidence rates. It is quite challenging for now and the future based on the huge population of China. The outcomes of breast cancer have been consistently improved and the current 5-year survival rate reaches 73%^17^. Therefore, the quality of life (QOL) of the long-term breast cancer survivors is more and more important. According to the previous studies, overweight and obesity are risk factors in patients with breast cancer^18^. Despite few studies on the assessment of QOL on overweight and obese breast cancer survivors at present, the presentation of QOL of obese and normal weight survivors were not consistent among previous studies^19^. Some studies had investigated the relationship between body mass index (BMI) and QOL, and indicated that higher BMI was associated with poor QOL^20–24^. However, conflicting evidences have questioned the adverse influence of overweight and obesity on the QOL. Some research suggested that within people with chronic disease(s), the ones with overweight or obesity had better QOL than the ones without these^25–27^. Therefore, the aim of this study was to investigate the association between BMI and QOL in breast cancer survivors in China.

## Materials and Methods

### Subjects

The present study was a population-based cross-sectional study. Breast cancer survivors were recruited from the affiliated groups of Cancer Rehabilitation Clubs in 34 cities across China. Exclusion criteria involved: severe physical and mental comorbidity, being illiterate, and missing BMI data. Approval for the research protocol was received from the Ethic Committee of Public Health School of Fudan Univeristy (protocol number RB # 2013-04-0450). A written Informed Consent was obtained from each participant prior to participation in the study and all methods were performed in accordance with the guidelines and regulations.

A self-completed questionnaire was used to collect information of participants including birthday, height, weight, marital status, educational level, financial, tumor stage, time since diagnosis, treatment, exercise, chronic conditions, and their use of tobacco and alcohol, QLO-C30 and QLQ-BR23. The chronic diseases included hypertension, hyperlipidemia, hyperuricemia, type I and II diabetes mellitus, coronary heart disease, stroke, respiratory disease, digestive system disease, and musculoskeletal disorders, which were collected from the participants self-reported results. Inclusion criteria were met by 10708 of the 10794 breast cancer survivors.

### Measurement of QOL

The simplified Chinese version of the Quality of Life Questionnaire-Core 30 items (EORTC QLQ-C30) and the Breast Cancer-specific module QLQ-23 also developed by the EORTC were used as the measures of QOL. The QLQ-C30^28^ encompassed five functional scales (Physical, Role, Cognitive, Emotional, and Social Functioning); three symptom scales (Fatigue, Pain, and Nausea/Vomiting); and a Global Health Status/QOL scale. Six single item scales were also included (Dyspnoea, Insomnia, Appetite Loss, Constipation, Diarrhoea, and Financial Difficulties). The EORTC QLQ-BR23^29^ incorporated two functional scales (Body image and Sexual Function) and three symptom scales (Systemic therapy side effects, Arm and Breast symptoms). Three single item scales (Upset by hair loss, Future respective, and Sexual enjoyment) were also included. A 4-point response scale - except for the global health status which were rated on a 7-point scale from 1 (very poor) to 7 (excellent), was used (1 = not at all; 4 = very much). The time frame was “during the past week”, except for the sexual functioning and sexual enjoyment (“during the past four weeks”). Scale scores were calculated by averaging items within scales and transforming average scores linearly into a 0 to 100 scale. A high score for a functional scale represented a high/healthy level of functioning whereas a high score for a symptom scale or item represented a high level of symptomatology or other problems. More details on the scoring procedures could be found in the EORTC QLQ-C30 Scoring Manual^30^. The psychometric properties of the questionnaire were tested and in conclusion it was found to possess the required standards such as validity (measuring what it is intended to measure), reliability (measuring with sufficient precision) and sensitivity (ability to detect changes)^31^.

### Categorization of BMI

In this study, BMI, a person’s weight (in kilograms) divided by the square of his or her height (in metres), was divided into four categories according to the WHO guidelines:^32^ underweight (<18.5 Kg/m^2^), normal weight (18.5–24.9 Kg/m^2^), overweight (25.0–29.9 Kg/m^2^) and obesity (≥30 Kg/m^2^).

### Data Analysis

All statistical analyses were performed using the Statistical Analysis System (SAS, Version 9.4). Participants’ characteristics and QOL were summarized by ratios and percentages for categorical variables and mean (standard deviation) for continuous variables. Differences in means for continuous variables were compared using analysis of variance (ANOVA), and differences in proportions were tested by ${\chi }^{2}$ test. Standard least squares regression was used to evaluate the independent association between BMI and QOL. Bonferroni test was applied to analyze the difference between groups. Statistical inferences were two-sided and P value less than 0.05 were considered as statistically significant.

## Results

### Clinical Characteristics

Among 10794 breast cancer survivors, 10708 subjects met the inclusion criteria. Table 1 summarized the clinical characteristics of the 10708 breast cancer survivors who were included in the analysis. The proportions of the participants were 3.42%, 67.90%, 25.60% and 3.08% for the underweight, normal weight, overweight and obese respectively based on the international BMI categories. The mean (SD) age and BMI were 56.99 (5.50) years and 23.63 (3.20) Kg/m^2^. The characteristics of the subjects didn’t equally distribute among the BMI categories (P < 0.05), except for marital status, financial, tumor stage and surgery. A majority (70.85%) of the participants had one or more comorbid conditions. Subjects with excess weight were more likely to suffer from chronic diseases, relapse, metastasis, and tended to drink and smoke more. Obesity was more frequent in subjects who never exercised.

**Table 1:** Demographic and Clinic Characteristics.

	Total (N = 10708)	Underweight (N = 366)	Normal (N = 7271)	Overweight (N = 2741)	Obesity (N = 330)	P
Age, mean(SD), (yr)	56.99 (5.50)	56.41 (6.36)	56.94 (5.50)	57.19 (5.45)	57.20 (5.92)	0.030
BMI, mean (SD)	23.63 (3.20)	17.36 (1.07)	22.35 (1.65)	26.79 (1.26)	32.67 (3.74)	<0.001
Married/cohabitating	88.46	84.15	88.43	89.09	88.48	0.052
Employment	6.85	8.47	7.58	5.03	3.94	<0.001
Income	22.53	21.58	23.35	20.76	20.30	0.090
Years since diagnosis	7.38 (5.95)	7.51 (5.77)	7.37 (5.89)	7.39 (6.09)	7.55 (6.29)	0.722
Tumor stage						0.052
Stage I	38.71	37.86	39.94	35.89	33.33	
Stage II	16.42	12.14	16.41	16.88	18.30	
Stage III	37.97	42.23	37.14	39.79	37.25	
Stage IV	6.91	7.77	6.51	7.44	11.11	
Surgery	98.37	98.55	98.44	98.44	97.58	0.313
Radiotherapy	46.08	41.28	45.36	46.20	52.39	0.001
Chemotherapy	91.43	87.74	91.22	92.11	91.60	0.034
Endocrine therapy	64.19	61.10	64.42	65.13	60.07	0.030
Relapse	7.24	9.78	6.72	7.99	9.66	0.011
Metastasis	7.16	8.10	6.78	7.58	11.08	0.019
Drinking	7.76	6.15	7.04	9.16	9.21	0.008
Smoking: Never	96.78	97.53	97.14	96.07	93.90	0.002
Former	2.39	2.47	2.12	2.90	3.96	
Current	0.84	0.00	0.75	1.03	2.13	
Exercise	75.56	72.73	75.92	76.05	66.77	0.001
Sleeps	19.33	25.82	19.51	17.90	19.94	<0.001
Comorbidity						<0.001
0	29.15	39.34	32.68	20.36	13.03	
1	24.57	33.06	26.02	20.43	17.58	
2	19.28	15.03	18.83	20.87	20.91	
≥3	27.00	12.57	22.47	38.34	48.48	
Hypertension	26.57	10.77	22.21	37.70	48.30	<0.001
Hypertriglyceridemia	31.47	17.08	27.48	42.07	48.46	<0.001
Hyperuricemia	7.26	3.32	5.90	10.65	13.79	<0.001
Type I diabetes	4.13	1.94	3.60	5.75	5.23	<0.001
Type II diabetes	8.27	2.23	7.11	11.61	13.50	<0.001
CHD	11.51	6.91	10.16	14.67	20.50	<0.001
Stroke	3.31	1.94	3.11	3.61	6.96	<0.001
Respiratory system	9.21	10.17	8.85	9.22	16.03	0.003
H & G	46.38	39.12	43.19	54.33	59.19	<0.001
Musculoskeletal	24.43	18.28	22.11	30.10	36.36	<0.001

1) Abbreviation: CHD, Coronary heart disease; H & G, Hepatobiliary & Gastrointestinal system;

2) Comorbidity: Sum of the total chronic diseases survivors suffered from.

### Relationship between BMI and QOL

Overweight breast cancer survivors reported significantly (P < 0.05) better QOL in almost all domains compared with underweight, normal weight and obese ones (Table 2). Overweight breast cancer survivors had significantly better QOL than normal weight ones in domains of global health status, emotional function, social function, cognitive function, fatigue, nausea and vomiting, insomnia, appetites loss, constipation, diarrhea, systemic therapy side effects, and arm symptoms (P < 0.05). The obese breast cancer survivors had a similar QOL with normal weight ones in almost all scales (P > 0.05) except for domains of dyspnoea and arm symptoms (P < 0.05). Compared with the normal weight breast cancer survivors, the underweight ones had significantly (P < 0.05) lower QOL in the domains of global health status, physical function, fatigue, pain, dyspnoea, insomnia, and appetites loss. In domains of role function, sexual function, sexual enjoyment, body image, future respective, breast symptom and upset by hair loss, no significant (P > 0.05) differences were found among the four BMI groups. The mean scores of the QOL increased with the increasing BMI from the underweight to the overweight in function scales, but decreased in symptom scales.

**Table 2:** QOL of breast cancer survivors according to BMI categories. Mean

	Adjusted scores				P	Differences between normal					
	Underweight	Normal	Overweight	Obesity		Underweight		Overweight		Obesity	
						t	Pr > |t|	t	Pr > |t|	t	Pr > |t|
**Function scores**											
**Scales covered by QLQ-C30**											
QL	61.32	65.61	67.57	65.37	<0.001	−3.48	0.003	3.64	0.002	−0.19	1.000
PF	81.54	84.62	85.00	82.75	<0.001	−4.31	<0.001	1.24	1.000	−2.45	0.086
EF	82.93	83.76	85.49	84.24	<0.001	−0.90	1.000	4.35	<0.001	0.49	1.000
SF	80.51	81.00	82.91	80.84	0.002	−0.41	1.000	3.71	0.001	−0.12	1.000
RF	88.31	90.35	90.90	90.66	0.064						
CF	78.14	80.32	81.48	79.99	0.002	−2.26	0.142	2.79	0.032	−0.32	1.000
**Scales covered by QLQ-BR23**											
BRSEF	93.11	92.67	92.68	92.25	0.884						
BRSEE	91.62	91.69	92.00	90.43	0.528						
BRBI	66.60	67.33	67.76	66.04	0.607						
BRFU	61.89	61.48	62.26	61.35	0.766						
**Symptom scores**											
**Scales covered by QLQ-C30**											
FA	31.67	25.91	23.63	23.42	<0.001	5.86	<0.001	−5.34	<0.001	−2.37	0.107
NV	4.65	3.35	2.68	2.51	<0.001	2.38	0.105	−2.83	0.028	−1.42	0.926
PA	19.09	15.75	15.36	17.09	<0.001	3.58	0.002	−0.97	1.000	1.34	1.000
DY	16.16	13.33	13.70	17.17	<0.001	2.75	0.036	0.82	1.000	3.49	0.003
SL	22.54	19.53	17.41	19.92	<0.001	2.71	0.040	−4.44	<0.001	0.32	1.000
AP	12.82	7.78	5.87	7.36	<0.001	5.70	<0.001	−4.99	<0.001	−0.44	1.000
CO	12.13	10.36	8.29	8.68	<0.001	1.73	0.498	−4.68	<0.001	−1.53	0.755
DI	10.09	7.86	6.63	7.81	<0.001	2.57	0.061	−3.25	0.007	−0.05	1.000
FI	30.20	28.39	28.16	32.26	0.099						
**Scales covered by QLQ-BR23**											
BRST	17.99	16.76	15.88	16.47	0.004	1.80	0.432	−2.98	0.017	−0.40	1.000
BRBS	17.73	17.33	16.64	16.05	0.208						
BRAS	21.76	22.16	23.87	25.88	<0.001	−0.36	1.000	3.55	0.002	3.14	0.010
BRHL	20.26	19.22	18.51	18.84	0.618						

1) Abbreviations: QL, global health status; PF, physical function; EF, Emotional function; SF, Social function; RF, Role function; CF, Cognitive function; BRST, systemic therapy side effects; BRSEF, sexual function; BRSEE, sexual enjoyment; BRBI, body image; BRFU, future respective; FA, fatigue; NV, nausea and vomiting; PA, pain; DY, dyspnoea; SL, insomnia; AP, appetites loss; CO, constipation; DI, diarrhoea; FI, financial difficulties; BRBS, breast symptoms; BRAS, arm symptoms; BRHL, upset by hair loss.

2) Scores of QOL was analyzed by standard least squares adjustment for age, educational level, number of chronic diseases, marital status, residence conditions, occupation, relapse, transfer, exercise, use of alcohol and tobacco, breakfast, vegetable, fruits and sleeps condition;

3) Analysis of Variance was used to get the P Value. Bonferroni test was applied to analyze the difference between groups.

### Relationship between BMI and QOL stratified by status of chronic conditions

Mean scores of the reported QOL in breast cancer survivors according to BMI categories and status of chronic conditions were described in Table 3. The covariates which the present study chose to adjust included age, educational level, number of chronic diseases, marital status, occupation, relapse, transfer, exercise, use of alcohol, breakfast diet, vegetables and fruits consumption as well as tobacco use. In the group of breast cancer survivors with no chronic disease, the QOL increased gradually along with BMI in the groups ranged from the underweight to the overweight in almost all domains; obese breast cancer survivors reported significantly worse QOL compared with normal weight and overweight ones; Overweight breast cancer survivors reported significantly higher QOL in domains of emotional function and fatigue, and had similar scores in all other domains when compared with normal weight ones; Underweight breast cancer survivors had significantly worse QOL in domains of constipation and diarrhea compared with normal weight ones.

**Table 3:** QOL of breast cancer survivors by BMI categories and status of chronic conditions. Mean.

	Adjusted scores				P	Differences between normal					
	Underweight	Normal	Overweight	Obesity		Underweight		Overweight		Obesity	
						t	Pr > |t|	t	Pr > |t|	t	Pr > |t|
**No Chronic Disease (N** = **3121)**											
**Function scores**											
**Scales covered by QLQ-C30**											
QL	65.90	68.96	70.38	55.63	<0.001	−1.58	0.691	1.29	1.000	−3.92	<0.001
PF	86.17	87.39	88.10	84.12	0.103						
EF	84.33	86.12	88.38	77.21	<0.001	−1.24	1.000	2.78	0.033	−3.48	0.003
SF	84.27	83.18	85.49	73.28	0.001	0.60	1.000	2.27	0.141	−3.04	0.014
RF	91.12	91.64	92.39	91.06	0.742						
CF	81.09	84.10	85.42	80.57	0.020	−2.09	0.218	1.63	0.620	−1.39	0.995
**Scales covered by QLQ-BR23**											
BRSEF	92.94	89.96	91.50	86.69	0.019	2.09	0.218	2.00	0.270	−1.32	1.000
BRSEE	92.54	88.63	90.90	86.27	0.015	2.16	0.185	2.40	0.099	−0.77	1.000
BRBI	71.83	71.57	73.48	65.57	0.148						
BRFU	67.22	66.43	67.37	64.36	0.880						
**Symptom scores**											
**Scales covered by QLQ-C30**											
FA	24.14	20.86	18.06	24.49	<0.001	2.17	0.181	−3.29	0.006	1.34	1.000
NV	4.76	3.45	2.15	3.15	0.028	1.43	0.917	−2.51	0.074	−0.18	1.000
PA	12.68	11.65	11.04	18.35	0.026	0.76	1.000	−0.81	1.000	2.78	0.033
DY	10.92	9.03	9.79	17.08	0.009	1.31	1.000	0.93	1.000	3.13	0.011
SL	16.08	14.63	12.74	19.14	0.035	0.91	1.000	−2.12	0.205	1.59	0.670
AP	9.25	6.52	4.94	6.97	0.018	2.09	0.220	−2.15	0.189	0.197	1.000
CO	11.39	7.59	5.59	10.86	<0.001	2.77	0.033	−2.61	0.055	1.33	1.000
DI	9.25	5.18	3.73	11.28	<0.001	3.47	0.003	−2.19	0.173	2.94	0.020
FI	26.25	26.71	25.97	43.13	0.006	−0.18	1.000	−0.50	1.000	3.41	0.004
**Scales covered by QLQ-BR23**											
BRST	14.28	12.83	12.49	17.94	0.017	1.40	0.978	−0.58	1.000	2.78	0.033
BRBS	11.04	13.71	13.40	18.84	0.030	−1.98	0.285	−0.42	1.000	2.14	0.193
BRAS	16.75	17.51	18.42	27.05	0.006	−0.48	1.000	1.02	1.000	3.39	0.004
BRHL	19.76	17.13	16.41	25.42	0.109						
**At least One Chronic Disease (N** = **7587)**											
**Function scores**											
**Scales covered by QLQ-C30**											
QL	58.83	64.19	66.46	66.23	<0.001	−3.37	0.005	3.68	0.001	1.42	0.934
PF	79.11	83.47	83.81	81.88	<0.001	−4.65	<0.001	0.94	1.000	−1.88	0.365
EF	82.37	82.78	84.44	84.65	0.002	−0.35	1.000	3.63	0.002	1.74	0.487
SF	78.43	80.07	81.98	81.52	0.007	−1.05	1.000	3.16	0.009	1.03	1.000
RF	86.71	89.81	90.36	90.31	0.040	−2.49	0.077	1.15	1.000	0.45	1.000
CF	76.97	78.75	79.94	79.00	0.034	−1.41	0.951	2.42	0.092	0.21	1.000
**Scales covered by QLQ-BR23**											
BRSEF	92.83	93.82	93.17	93.72	0.288						
BRSEE	90.56	92.99	92.66	91.78	0.184						
BRBI	64.21	65.61	65.61	64.89	0.842						
BRFU	59.58	59.42	60.27	59.61	0.813						
**Symptom scores**											
**Scales covered by QLQ-C30**											
FA	35.30	27.98	25.85	24.63	<0.001	5.72	<0.001	−4.31	<0.001	−2.90	0.023
NV	4.68	3.30	2.82	2.40	0.021	2.01	0.267	−1.82	0.409	−1.45	0.883
PA	22.42	17.47	17.03	17.78	<0.001	4.01	<0.001	−0.91	1.000	0.28	1.000
DY	18.77	15.14	15.31	18.18	0.006	2.63	0.051	0.33	1.000	2.46	0.085
SL	25.58	21.50	19.44	21.43	<0.001	2.77	0.033	−3.66	0.002	−0.05	1.000
AP	14.96	8.29	6.30	7.73	<0.001	5.71	<0.001	−4.43	<0.001	−0.53	1.000
CO	11.83	11.51	9.40	9.10	<0.001	0.23	1.000	−3.93	0.001	−1.92	0.333
DI	10.13	8.97	7.75	7.89	0.027	0.98	1.000	−2.67	0.046	−1.01	1.000
FI	32.49	29.11	28.90	30.99	0.299						
**Scales covered by QLQ-BR23**											
BRST	19.61	18.40	17.30	17.15	0.003	1.35	1.000	−3.19	0.009	−1.55	0.728
BRBS	21.09	18.84	18.00	16.41	0.011	1.83	0.402	−1.76	0.474	−2.19	0.173
BRAS	23.92	24.08	25.94	26.72	0.005	−0.11	1.000	3.24	0.007	1.98	0.289
BRHL	20.42	20.12	19.31	18.08	0.537						

1) Abbreviations: QL, global health status; PF, physical function; EF, Emotional function; SF, Social function; RF, Role function; CF, Cognitive function; BRST, systemic therapy side effects; BRSEF, sexual function; BRSEE, sexual enjoyment; BRBI, body image; BRFU, future respective; FA, fatigue; NV, nausea and vomiting; PA, pain; DY, dyspnoea; SL, insomnia; AP, appetites loss; CO, constipation; DI, diarrhoea; FI, financial difficulties; BRBS, breast symptoms; BRAS, arm symptoms; BRHL, upset by hair loss.

2) Scores of QOL was analyzed by standard least squares, in None of the Chronic disease group, adjustment for age, educational level, marital status, residence conditions, occupation, relapse, transfer, exercise, use of alcohol and tobacco, breakfast, vegetable, fruits and sleeps condition; in combined with chronic diseases group, adjustment for the above factors and number of chronic diseases.

3) Analysis of Variance was used to get the P Value. Bonferroni test was applied to analyze the difference between groups.

In the group of breast cancer survivors with one or more chronic diseases, the QOL increased gradually along with BMI in the groups ranged from underweight to obese in almost all scales. Compared with the normal weight breast cancer survivors group, the overweight group had significantly higher QOL in domains of global health status, emotional function, social function, fatigue, insomnia, appetites loss, constipation, diarrhoea, systemic therapy side effects, and arm symptoms; and the underweight group had significantly lower QOL in global health status, physical function, fatigue, pain, insomnia and appetites loss. The obese breast cancer survivors had similar QOL with normal weight ones in all scales (P > 0.05) except in domains of fatigue (P < 0.05).

The counterintuitive relationship between BMI and QOL was similar in the groups of breast cancer survivors without chronic diseases and in those with one or more chronic diseases. Nevertheless, the effect was more pronounced in the group of breast cancer survivors who had one or more chronic diseases.

## Discussion

Contemporarily, innovative clinical treatments have greatly improved the survivals of breast cancer, making QOL the next important requirement of survivors. QOL has received extensive attention as an outcome measurement in public health and clinical medicine. In the present study, QOL of breast cancer survivors was measured by EORTC QLQ-C30 and EORTC QLQ-BR23. Although certain clinical factors^33^ and socioeconomic status^34^, such as stage, age, income, marital status *et al*. are considered to be the most important factors influencing survivals of breast cancer, these are factors which can’t be modified. In contrast, body weight was one of the few potentially modifiable lifestyle factors. Therefore, the goal of this study was to explore the association between BMI and QOL in Chinese women with breast cancer.

Since chronic diseases were important determinant of QOL^35^ and BMI was associated with chronic diseases^36,37^, we divided two groups to determine the association between BMI and QOL according to whether or not comorbidities existed. Different patterns were observed in breast cancer survivors with and without chronic diseases. The symptom scores seemed to be more impacted than function scores, which might be due to the defensive function of fat. In some domains of the significant increases of QOL in overweight group, the absolute values of score increments were less than 5 points. Osoba *et al*^38^. recommended that a difference of more than 5 points on the 0–100 scale were considered as a clinically important difference. Maringwa JT *et al*^39^. suggested that 5 to 10 units of the QLQ-C30 scales might be used as guidance for clinicians and researchers to classify patients as improved or deteriorated in QOL and symptoms over time, and hence to determine the proportion of patients benefiting from treatment. Since we did not regard BMI as a clinical treatment for the breast cancer survivors, we think the differences in the QOL score in BMI categories still had great significance for readers and should be aware of. It may be useful in directing intervention efforts for improving QOL.

For the aspects of overweight and obesity, people usually associate them with worse outcomes, while their prevalence increasing worldwide. There had been numerous studies^40–42^ which reported that overweight and moderately obese patients had a lower mortality rate and a better survival. This counterintuitive result - a new review of being overweight or obese - was called “obesity paradox”^43^. In the previous studies of BMI and QOL, the “obesity paradox” phenomenon has been found existed in healthy people^44–46^ and people with chronic diseases^46–48^. Our results suggest that the “obesity paradox” phenomenon also existed in breast cancer survivors in China. The positive influence of being overweight on QOL in breast cancer survivors remains somewhat debatable due to specific limitations in the present study. Nutritional factors might contribute to this positive effect. Diseases and treatments could weaken the patients’ strength and make them more fragile, while overweight patients might have certain resistance to this. On the other hand, obese people were less likely to suffer from depressive symptoms compared with those with normal weight^49^, which supported the “liberal mind brings health” or “jolly fat” hypothesis. A positive attitude could also be beneficial to QOL. Although a series of hypotheses had been put forward to explain the “obesity paradox”^50^, the underlying reasons and the biological mechanisms for the increased QOL among overweight and even obese breast cancer survivors still remained elusive. Further research was needed to explore the reasons for these phenomena.

We also found that: underweight impaired the QOL of breast cancer survivors, which was consistent with previous researches^46,51–53^. Although our results showed that obese breast cancer survivors with one or more chronic diseases had a similar QOL compared with normal weight ones in almost all scales, the overall scores decreased significantly, which was consistent with other studies which included that chronic diseases could impair QOL^35^. Considering obesity was the confirmed risk factor for chronic diseases, being slightly overweight is recommended for breast cancer survivors.

Some limitations of this study should be addressed. This study was a cross-sectional survey and the participants might only represent for the breast cancer survivors in China. The BMI was calculated from the basis of breast cancer survivors self-reported body weight and height, which might cause certain errors and misclassifications. Nevertheless, the results of Dekkers *et al*. indicated that self-reported BMI was competently accurate to evaluate the prevalence of overweight/obesity in a middle-aged overweight working population^54^. The association between breast cancer and BMI was different in pre and post-menopausal women, as well as side effects of cancer treatment that affected QOL. Unfortunately, we did not collect the information of menopause in our questionnaire, we would like to carry out a more extensive study on the question in the future. In spite of these limitations, our results from large population-based study may be useful in directing intervention efforts for improving QOL and provide a reference and foundation for future researches.

## Conclusions

Through large population-based cross-sectional study, we observed significantly increasing trend of BMI associated with QOL for women with breast cancer in China. Overweight breast cancer survivors had a significantly better QOL than survivors with a BMI below 25 Kg/m^2^. Both underweight and obese breast cancer survivors showed impaired QOL, and further research focus should lie on them.
